# Effects of lutein supplementation on inflammatory biomarkers and metabolic risk factors in adults with central obesity: study protocol for a randomised controlled study

**DOI:** 10.1186/s13063-019-3998-8

**Published:** 2020-01-06

**Authors:** Juan Zhou, Dan Zhao, Ning Wang, Zhiwei Zeng, Changyi Wang, Liping Hao, Xiaolin Peng

**Affiliations:** 1 Shenzhen Nanshan Centre for Chronic Disease Control, Shenzhen, 518054 China; 2 Department of Nutrition and Food Hygiene, School of Public Health, Tongji Medical College, Huazhong University of Science and Technology, Wuhan, 430030 China; 3 Hubei Key Laboratory of Food Nutrition and Safety, School of Public Health, Tongji Medical College, Huazhong University of Science and Technology, Wuhan, 430030 China; 4 Department of Non-communicable Disease Prevention and Control, Shenzhen Nanshan Center for Chronic Disease Control, 7 Hua Ming Road, Shenzhen, 518054 China

**Keywords:** Lutein supplementation, Central obesity, Inflammatory biomarkers, Metabolic risk factors

## Abstract

**Background:**

The prevalence of central obesity is constantly increasing, and visceral fat is associated with increased production of inflammatory factors and metabolic risk factors. Lutein might retard the development of metabolic disease through its antioxidant and anti-inflammatory properties. Furthermore, epidemiological studies have associated higher dietary intake and serum levels of lutein with decreased adiposity. However, few randomised controlled trials have shown the effects of lutein supplementation on inflammatory biomarkers and metabolic risk factors, especially in adults with central obesity.

**Methods:**

This study will be conducted as a double-blind, parallel placebo-controlled clinical trial in which 120 people who have central obesity, are 18 to 60 years old and are willing to provide informed consent will be randomly assigned to the intervention or placebo group in a 1:1 ratio according to sex, age and waist circumference. The intervention group will receive 10 mg daily lutein supplementation for 12 weeks to explore the effect of lutein supplementation on serum lutein, glycaemic and lipid profiles, inflammatory factors and body composition. Two populations (intention-to-treat population and per-protocol population) will be used in the data analyses.

**Discussion:**

Our findings from this trial will contribute to the knowledge of the association between lutein supplementation and inflammatory biomarkers and metabolic risk factors in people with central obesity and will offer a possibility for the prevention of inflammatory diseases.

**Trial registration:**

Chinese Clinical Trial Registry: ChiCTR1800018098. Registered on 30 August 2018.

## Introduction

The prevalence of obesity has constantly increased and has reached epidemic proportions. The World Health Organization estimated that, by 2016, 39% of adults 18 years or over were overweight and 13% were obese worldwide [1]. Obesity represents a major risk factor for an expanding set of chronic diseases, such as type 2 diabetes mellitus, hypertension, cardiovascular diseases and cancer [2–4]. Specifically, central obesity, which occurs when excessive abdominal fat is deposited around the stomach and abdomen, is considered to be the most detrimental type of obesity and is accompanied by an increased risk of mortality [5, 6]. Substantial studies have suggested that oxidative stress and inflammation may be mechanistic links between obesity and metabolic diseases [7].

It is clear that the levels of oxidative stress and inflammatory factors increase as the amount of adipose tissue increases. The adipokines—interleukin-6 (IL-6), tumour necrosis factor-alpha (TNF-α), adiponectin, leptin and resistin—secreted by adipose tissue play a role in the homeostasis of various physiological processes, such as insulin sensitivity, energy expenditure and fatty acid oxidation [8, 9]. However, compared with other fat deposits, visceral fat has more adverse effects, including higher levels of free fatty acids, inflammatory molecules, and adipocytokines [10–13]. Epidemiological studies have indicated that visceral fat is correlated with insulin resistance (IR) and glucose intolerance and increasing levels of C-reactive protein (CRP), TNF-α, IL-6, isoprostanes and monocyte chemoattractant protein-1 (MCP-1) [13, 14].

Lutein is an oxygenated carotenoid that is found primarily in dark green leafy vegetables and egg yolks. Lutein is known mostly for its effects on visual function and its preventative effect against macular degeneration, potentially acting as an antioxidant that protects against light-induced oxidative damage in the retina by oxygen radicals [15, 16]. Given the beneficial antioxidant and anti-inflammatory properties [16], it is hypothesised that lutein may have beneficial effects on inflammatory biomarkers and metabolic risk factors. Xu et al. [17] provided lutein supplementation to patients with early atherosclerosis and found that the intervention group had significant decreases in serum IL-6 and MCP-1. An intervention study conducted in healthy non-smokers revealed that lutein supplementation increased total antioxidant capacity and reduced CRP [18]. Although accumulating epidemiological studies have shown an inverse relationship between serum carotenoid concentrations and circulating markers of inflammation, such as CRP and soluble intercellular cell adhesion molecule-1 (ICAM-1), intervention studies have focused mostly on other carotenoids, such as lycopene [19, 20], and less on lutein.

Another hypothesis is that lutein may have anti-adiposity effects. Our previous study found that lutein treatment significantly decreased body weight and abdominal and total adipose tissue, which were increased by high-fat-diet feeding in mice [21]. In addition, the concentrations of total cholesterol (TC) and triglycerides (TGs) in the serum and liver and the serum levels of low-density lipoprotein cholesterol (LDL-C) were significantly reduced [21]. Also, epidemiological studies have associated higher dietary intakes and serum levels of lutein with decreased adiposity [22–24]. However, despite animal studies and human epidemiological studies linking lutein to adiposity, there is a paucity of randomised controlled trials (RCTs) using lutein supplementation to assess changes in adiposity.

An important aspect to consider with lutein supplementation is that it cannot be synthesised by humans and can be obtained only through diet or supplements. However, our previous study revealed that the median intake of lutein (2.499 mg/d) is far below the special proposed level (10 mg/d) that is recommended in the 2013 Chinese Dietary Reference intakes [25]. In addition, although the antioxidant properties of lutein have been proven in different populations, reports dealing specifically with lutein in people with central obesity are scarce. In light of the information above, providing lutein supplementation to people with central obesity is worthy of exploration.

## Methods

### Study design and setting

**Fig. 1:**
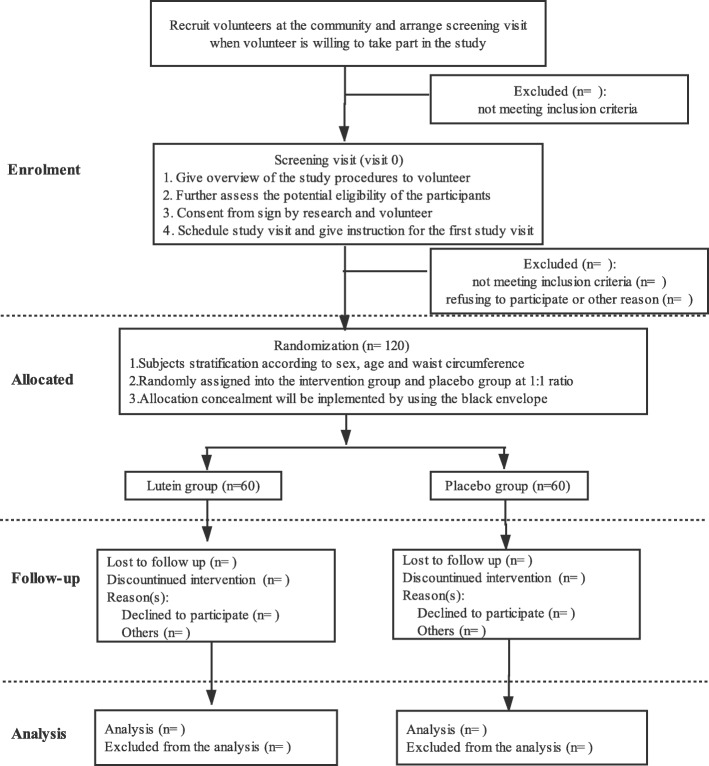
Flowchart of the trial

This is a double-blind, parallel RCT enrolling participants with central obesity. The Standard Protocol Items: Recommendations for Interventional Trials (SPIRIT) 2013 statement will be followed in this trial (Additional file 1: SPIRIT checklist) [26]. The proposed clinical trial will be conducted at the Community Health Service Centre (CHSC) in Nanshan District, Shenzhen, Guangzhou Province, China, for 12 weeks to assess the effects of lutein supplementation (10 mg/d) on inflammatory biomarkers and metabolic risk factors in adults with central obesity. Figure 1 illustrates the overview of the study.

### Study participants and eligibility criteria

It is estimated that 52 participants per treatment group would be required for 80% power and a 5% level of significance to detect the change in serum TGs over 12 weeks of supplementation [27]. Allowing for a dropout rate of about 15%, we aim to recruit 120 participants in total. Participants were eligible for this study if they fulfilled the following criteria: (1) age between 18 and 60 years, (2) waist circumference (WC) of at least 90 cm for males and at least 85 cm for females, (3) deficient lutein intake (dietary lutein intake of less than 10 mg/d), and (4) willingness to sign the informed consent form. People meeting any of the following criteria will be excluded from participation: (1) use of lutein supplementation or related health-care products within 1 month, (2) use of antibiotics within 1 month, (3) secondary obesity, (4) adoption of a special diet or meal replacement in the previous 2 weeks, (5) use of insulin or insulin sensitizers within 1 month, (6) chronic diseases (including type 2 diabetes, hypertension and dyslipidaemia) for over 10 years, (7) hepatorenal dysfunction (creatinine of more than 1.2 times the upper normal limit of creatinine, aspartate aminotransferase (AST) of more than 1 time the upper normal limit), (8) any active tumours/cancers, (9) regular use of drugs affecting blood lipid, (10) pregnancy or lactation or (11) history of gastrointestinal surgical operation.

### Randomisation and blinding

After the inclusion and exclusion criteria are assessed and informed consent is obtained, participants will be allocated to the lutein group or the placebo group. Computer-generated random numbers will be used for allocation sequence generation and stratification will be completed according to sex, age and WC. This part of the study will be performed by a person who is operationally independent from the study team. Allocation concealment will be implemented by using a random number card in a black envelope. The details of the allocation will be concealed from the investigators, outcome assessors and the patients until all data collection is complete. The collected data of the patients will be kept confidential during this trial.

### Steering committee and monitoring

The steering committee consists of JZ, DZ, NW, ZW-Z, CY-W, LP-H and XL-P. The steering committee is responsible for the design, reporting and publication of the trial. Clinical trial monitoring will be performed by an independent monitor who will check reports and documents. The frequency of monitoring can be adjusted in accordance with the total enrolment period and enrolment rate.

### Supplements

The participants will receive either 10 mg of lutein or placebo capsules per day for 12 weeks. Lutein (10 mg) will be mixed with pregelatinous starch (240 mg) and provided in acid-soluble gelatine capsules, and the placebo will contain pregelatinous starch (250 mg). To maintain and guarantee blinding, the capsules will be identical in appearance, shape, smell and weight. All capsules will be provided by the Angel Nutrition Co., Ltd. (Yichang, China). Face-to-face instruction on how to take the capsules will be provided at baseline. The enrolled participants will be required to (1) take one capsule along with meals daily; (2) avoid making any changes to dietary habits, physical activity level (PAL) or medicine use; and (3) record changes in dietary habits, PAL and medicine use in a timely manner.

### Study procedures

#### Recruitment and screening

Table 1 provides the schedule for enrolment, intervention and follow-up. Participants will be recruited by placing advertisements on social media sites and community bulletin boards. Once a volunteer expresses initial interest in participating in the study, he or she will have face-to-face or telephone communication with the research assistant. Detailed information on the study trial, including the purpose, the methods of the intervention and duration, will be provided to the volunteers. If they are still interested in this project after careful consideration, the structured standardised screening questionnaire, which is based mainly on the inclusion and exclusion criteria for the study, will be used to assess the potential eligibility of the participants for the study. Potential participants who are found not to be eligible will be excluded at this stage. Eligible participants will be invited to attend a further screening visit, which will take place at the CHSC. First, informed consent will be obtained from all participants. Second, for safety assessments, liver and renal function will be evaluated by collecting fasting blood samples and spot urine samples. Third, the anthropometric index will be measured by the trained investigators.

**Table 1:** Schedule for screening, intervention and follow-up (test visit)

	Screening (−4 week)	Test visit 1 (week 0)	Test visit 2 (week 6)	Test visit 3 (week 12)
Screening questionnaire	X			
Demographic data	X			
Medication, health status and any changes	X	X	X	X
Informed consent	X			
Height and weight	X	X	X	X
Waist circumference and hip circumference	X	X	X	X
Body composition (bioelectric impedance)	X	X	X	X
Blood pressure	X	X	X	X
Blood sample	X	X	X	X
Allocation		X		
Physical activity questionnaire		X	X	X
Mini-mental State Examination		X		X
24-h diet recall		X	X	X
Spot urine sample	X	X		X
Faecal sample		X		X
Provided supply of capsules		X	X	
Tablet count			X	X
Compliance assessment			X	X
Assess adverse reactions			X	X
Collect unused test product			X	X

**Table 2:** Biological and anthropometric parameters analysed in the lutein supplementation study

	Variable	Screening (−4 week)	Test visit 1 (week 0)	Intermediate visit (week 6)	Test visit 2 (week 12)
Biological	TC	X	X	X	X
	TGs	X	X	X	X
	HDL-C	X	X	X	X
	LDL-C	X	X	X	X
	ALT	X	X	X	X
	AST	X	X	X	X
	Creatinine	X	X	X	X
	FBG	X	X	X	X
	Insulin	X	X	X	X
	Lutein	X	X	X	X
	RBP4		X		X
	Adiponectin		X		X
	IL-6		X		X
	IL-1β		X		X
	CRP		X		X
	MCP-1		X		X
	TNF-α		X		X
Anthropometric measurement	WC	X	X	X	X
	HC	X	X	X	X
	BMI	X	X	X	X
	WHR	X	X	X	X
	WHtR	X	X	X	X
	BP	X	X	X	X

*Abbreviations*: *ALT* alanine aminotransferase, *AST* aspartate aminotransferase, *BMI* body mass index, *BP* blood pressure, *CRP* C-reactive protein, *FBG* fasting blood glucose, *HC* hip circumference, *HDL-C* high-density lipoprotein cholesterol, *IL* interleukin, *LDL-C* low-density lipoprotein cholesterol, *MCP-1* monocyte chemoattractant protein-1, *RBP4* retinol-binding protein 4, *TC* total cholesterol, *TG* triglyceride, *TNF-α* tumour necrosis factor-alpha, *WC* waist circumference, *WHR* waist-to-hip ratio, *WHtR* waist-to-height ratio

#### Test visit

At test visit 1 (week 0), eligible participants will be allocated to either a lutein supplement group or a control group. The lutein supplement group will take one 10 mg lutein capsule daily, and the control group will take the placebo. Participants will be given a 6-week supply of capsules at both visit 1 and visit 2 and will be required to return the remaining capsules at the next visit, which will be counted and recorded for each participant to ensure compliance with the protocol. Participants will be defined as non-compliant if they took less than 80% of the study product. Medication, health status or any recent changes around the inclusion and exclusion criteria since the completion of the screening questionnaires will also be re-assessed to evaluate whether participants are still suitable to participate in the study. Anthropometric measurements, dietary assessments and blood samples will be collected at each visit, and stool samples and spot urine samples will be collected at test visit 1 and test visit 3. At test visit 1 and test visit 3, the volunteers will be asked to complete the short form of the International Physical Activity Questionnaire (IPAQ-short form) and the Mini-mental State Examination (MMSE). At the end of each visit, participants will be thanked for their time and will be provided with breakfast. After test visit 3, people in the placebo group will be given the same amount of lutein supplements as that provided to the intervention group. The full details of the data that will be collected at each time point can be found in Table 1.

#### Anthropometric measurements

Anthropometric measurements will be collected by using standardised equipment and examination procedures. The weight and height of the participants will be measured while they are wearing light clothes and no shoes in the morning of the follow-up. Body fat percentage and total body water will be calculated by using a one-stop self-service monitoring machine (E-Techco Information Technologies Co., Shenzhen, China). WC will be measured at the midpoint between the lower edge of the rib arch and the iliac crest while the subjects are standing and breathing steadily. The hip circumference (HC) will be measured at the widest part of the buttocks at the intertrochanteric level to the nearest 0.1 cm. The other anthropometric indexes will be calculated by using the following equations: body mass index (BMI) = weight/height (kg/m^2^), waist-to-hip ratio (WHR) = waist/hip (m/m), and waist-to-height ratio (WHtR) = waist/height (m/m). Blood pressure (BP) will be measured by using a standard mercury sphygmomanometer with a cuff placed on the upper right arm after a 5-min rest with 1-min intervals before subsequent measurements. Three BP readings will be recorded, and the mean will be calculated. All anthropometric measures will be taken by trained research assistants using standard equipment in accordance with standard guidelines. A list of anthropometric measurements is presented in Table 2.

#### Biological sampling

At baseline and the middle and end of the trial, 12 mL of venous blood sample (4 mL in EDTA-coated sterile tubes and 8 mL in regular tubes) will be collected after the participants fast overnight. Table 2 summarises all the blood markers that are examined during the study. In addition to the indexes of liver and renal function (alanine aminotransferase (ALT), AST and creatinine), blood glucose, insulin, the lipid profile (TC, TGs, high-density lipoprotein cholesterol (HDL-C) and LDL-C) and CRP will be tested on the day of blood collection. Enzymatic methods (Biosino Biotechnology Company Ltd., Beijing, China) will be used for measuring serum hepatic enzymes, including ALT and AST. Serum creatinine will be determined by using the Jaffe method. Insulin concentrations (μU/mL) will be measured with a chemiluminescence microparticle immunoassay, high-sensitive CRP will be measured by using the immunoturbidimetry assay, and fasting blood glucose (mmol/L) will be measured by using the enzymatic colorimetric method with glucose oxidase using a commercially available kit. IR will be estimated by the homeostasis model assessment of insulin resistance (HOMA-IR): fasting insulin (μU/mL)*(fasting glucose (mmol/L)/22.5) [24]. The lipid profile will be determined by colorimetric methods using commercial kits (Biosino Biotechnology Company Ltd.) in a Hitachi 7080 automated analyser (Hitachi, Ltd., Tokyo, Japan). Serum lutein (μmol/L) will be analysed by high-performance liquid chromatography-tandem mass spectrometry. Retinol-binding protein 4 (RBP4) (μg/mL) and adiponectin (μg/mL) will be measured by using an enzyme-linked immunosorbent assay (ELISA) kit (Adipogen, San Diego, CA, USA). Serum levels of MCP-1, TNF-α, IL-6 and IL-1β will be measured by using the flow cytometry method (FCM).

Participants will be required to provide spot urine samples on the day of the screening and at test visit 1 and test visit 3. A routine urine test will be performed on the day of collection. Faecal samples will be collected at test visit 1 and visit 3 using a stool specimen collection kit, which includes the operating instructions, an ice pack, gloves, one sterile container and spoon, a sealed plastic pouch and a cool box. Participants will collect their faecal samples and bring them to the CHSC and then the samples will be transported and stored for further analysis.

A research biobank, where blood, urine and faecal samples will be stored with an identification number for later analysis of biomarkers, will be established in relation to this trial. Participants will be informed about the research biobank before signing the informed consent form. Only investigators and the research team will retain the right to access the relevant data.

#### Dietary assessments

At the screening periods, eligible participants will complete a questionnaire on food intake, which captures information on how many servings of vegetables rich in lutein they consume, including sweet potato leaves, spinach, broccoli, collards, leeks and lettuce. This questionnaire provides a reference to roughly evaluate dietary lutein intake. At each test visit, individuals will be required to provide 3 days of 24-h diet recall via the phone or the internet. The data will be collected and calculated by a professional nutritionist in accordance with the Chinese Food Composition Database (2009) and the US Department of Agriculture Nutrients Database [28, 29]. At each follow-up, questions on whether the participants have changed their diets will be asked, including questions regarding a significant reduction or increase in dietary intake.

#### Study outcomes

The primary outcome consists of changes in the inflammatory biomarkers between the intervention and placebo group from baseline (test visit 1) to 12 weeks later (test visit 2). The secondary objectives are to assess changes in the anthropometric indexes, including weight, WC, HC and body composition.

#### Data management and dissemination of results

Individuals’ data or other related information will be kept in a file located in the principal investigator’s office. The authorised study team will enter the information into the electronic database by using double-entry methods. The private personal information (e.g., ID number and telephone) will be hidden and restricted to the principal investigator. The electronic database will be unified and managed by the Shenzhen Nanshan Center for Chronic Disease Control. The results will be presented at scientific conferences as abstracts, oral presentations or posters. In addition, the results of the present study will be disseminated through publication in an international peer-reviewed journal.

#### Side effects and safety assessment

There are no side effects of 10 mg/d of lutein supplementation. An observed safe level (OSL) of 20 mg/d for lutein has been suggested [30]. There is no anticipated harm or compensation for participation in this study. If they feel uncomfortable, all participants can contact a research assistant at any time. All serious and non-serious adverse events or reactions involving capsule supplementation (or both) will be recorded by the research assistant. The allocated treatment may be performed without blinding or may be discontinued under special circumstances during the study if the investigator deems it necessary. If possible, the patients who discontinued or deviated from the protocol will be followed up regarding all endpoints.

### Statistical analysis

Two populations will be used in the analyses. The intention-to-treat (ITT) population will include all participants who have been randomly assigned, whereas the per-protocol (PP) population will include all participants who accomplish the entire intervention. The baseline characteristics of the study will be summarised as the means ± standard deviations for parametrically distributed data, geometric mean values (and 95% confidence intervals) for non-parametrically distributed data, and numbers (percentages) for categorical data.

Differences between participants who complete and withdraw from the trial will be analysed by using an independent *t* test or the Mann–Whitney test for continuous variables (e.g., age) and the chi-squared test for categorical variables (e.g., sex). For primary and secondary outcomes, analysis of covariance will be used to examine differences between lutein supplement and placebo at 12 weeks, adjusting for potential confounding factors and effect modifiers (e.g., baseline age and sex). The present study has no plan for subgroup analysis. Our statistical analysis will be performed by using R software, and the results will be considered significant at a *P* value of less than 0.05.

## Discussion

This study was designed to test the hypothesis that a lutein intervention will significantly modulate inflammatory factors and metabolic risk factors in people with central obesity compared with the effects observed in a control group receiving the placebo capsules. When this clinical study was designed, several key decisions were made to overcome the current limitations in the published literature. Previous studies on the antioxidant and anti-inflammatory properties of lutein focused mainly on the early atherosclerosis population [17, 31, 32]. However, as far as we are aware, well-controlled lutein supplementation is not common, and this study will be the only study of its type to be conducted in the context of central obesity. Central obesity is a state of chronic low-grade inflammation characterised by elevated concentrations of circulating inflammatory markers, which may be a critical link between metabolic disorders such as IR and diabetes [12, 33]. Therefore, individuals with central obesity also deserve attention.

Second, when focusing on central obesity, subjects with deficient lutein intake were considered. A larger body of research has indicated that dietary lutein intake or serum lutein levels (or both) are negatively associated with adiposity [22–24]. In addition, a previous longitudinal study found that obese subjects had lower concentrations of total carotenoids (including lutein) than subjects with a BMI of less than 22 kg/m^2^ [34]. These results indicate that central obesity may require more antioxidants to protect against oxidative stress and inflammation generated by adipose tissue.

However, we need to acknowledge that this clinical study has some limitations. First, the calculation of the dietary lutein intake is crude in the screening period and will be a reference for us to choose people who have a relatively lower intake. Second, this study will explore the short-term impact of the intervention because the participants will be treated for only 12 weeks. However, the development of metabolic diseases is a long and gradual process and can be influenced by other factors. Improvements in the inflammatory biomarkers and metabolic risk factors will not suggest that lutein intervention can prevent metabolic diseases; however, it can offer us a possibility for the prevention of inflammatory diseases. Whether lutein intervention can prevent metabolic diseases requires long-term intervention experiments.

## Trial status

The trial commenced recruitment in June 2019 and is currently open for recruitment. Recruitment will cease when 120 participants have been randomly assigned. It was anticipated that this target would be reached by October 2019.

## Supplementary information

**Additional file 1.** SPIRIT (Standard Protocol Items: Recommendations for Interventional Trials) 2013 Checklist: Recommended items to address in a clinical trial protocol and related documents.

## Data Availability

The datasets analysed during the present study are available from the corresponding author on reasonable request.

## Abbreviations

ALT: Alanine aminotransferase
AST: Aspartate aminotransferase
BMI: Body mass index
BP: Blood pressure
CHSC: Community Health Service Centre
CRP: C-reactive protein
HC: Hip circumference
IL: Interleukin
IR: Insulin resistance
LDL-C: Low-density lipoprotein cholesterol
MCP-1: Monocyte chemoattractant protein-1
PAL: Physical activity level
RCT: Randomised controlled trial
TC: Total cholesterol
TG: Triglyceride
TNF-α: Tumour necrosis factor-alpha
WC: Waist circumference

## References

[1] World Health Organization. Obesity and overweight. https://www.who.int/en/news-room/fact-sheets/detail/obesity-and-overweight. Accessed 20 June 2019.

[2] Hinnouho GM, Czernichow S, Dugravot A, Nabi H, Brunner EJ, Kivimaki M (2015). Metabolically healthy obesity and the risk of cardiovascular disease and type 2 diabetes: the Whitehall II cohort study. Eur Heart J.

[3] Lauby-Secretan B, Scoccianti C, Loomis D, Grosse Y, Bianchini F, Straif K (2016). Body fatness and cancer--viewpoint of the IARC working group. N Engl J Med.

[4] Leggio M, Lombardi M, Caldarone E, Severi P, D'Emidio S, Armeni M (2017). The relationship between obesity and hypertension: an updated comprehensive overview on vicious twins. Hypertens Res.

[5] Zhang C, Rexrode KM, van Dam RM, Li TY, Hu FB (2008). Abdominal obesity and the risk of all-cause, cardiovascular, and cancer mortality: sixteen years of follow-up in US women. Circulation.

[6] Katzmarzyk PT, Mire E, Bouchard C (2012). Abdominal obesity and mortality: the Pennington center longitudinal study. Nutr Diabetes.

[7] Marseglia L, Manti S, D'Angelo G, Nicotera A, Parisi E, Di Rosa G (2014). Oxidative stress in obesity: a critical component in human diseases. Int J Mol Sci.

[8] Liza K, Phillips JBP (2008). The link between abdominal obesity and the metabolic syndrome. Curr Hypertens Rep.

[9] Fernandez-Sanchez A, Madrigal-Santillan E, Bautista M, Esquivel-Soto J, Morales-Gonzalez A, Esquivel-Chirino C (2011). Inflammation, oxidative stress, and obesity. Int J Mol Sci.

[10] Ritchie SA, Connell JM (2007). The link between abdominal obesity, metabolic syndrome and cardiovascular disease. Nutr Metab Cardiovasc Dis.

[11] Hocking S, Samocha-Bonet D, Milner KL, Greenfield JR, Chisholm DJ (2013). Adiposity and insulin resistance in humans: the role of the different tissue and cellular lipid depots. Endocr Rev.

[12] Sam S, Haffner S, Davidson MH, D'Agostino RB, Feinstein S, Kondos G (2009). Relation of abdominal fat depots to systemic markers of inflammation in type 2 diabetes. Diabetes Care.

[13] Pou KM, Massaro JM, Hoffmann U, Vasan RS, Maurovich-Horvat P, Larson MG (2007). Visceral and subcutaneous adipose tissue volumes are cross-sectionally related to markers of inflammation and oxidative stress: the Framingham Heart Study. Circulation.

[14] Indulekha K, Anjana RM, Surendar J, Mohan V (2011). Association of visceral and subcutaneous fat with glucose intolerance, insulin resistance, adipocytokines and inflammatory markers in Asian Indians (CURES-113). Clin Biochem.

[15] Huang YM, Dou HL, Huang FF, Xu XR, Zou ZY, Lin XM (2015). Effect of supplemental lutein and zeaxanthin on serum, macular pigmentation, and visual performance in patients with early age-related macular degeneration. Biomed Res Int.

[16] Kijlstra A, Tian Y, Kelly ER, Berendschot TT (2012). Lutein: more than just a filter for blue light. Prog Retin Eye Res.

[17] Xu X-R, Zou Z-Y, Xiao X, Huang Y-M, Wang X, Lin X-M (2013). Effects of lutein supplementation om serum inflammatory cytokines, ApoE and lipid profiles in early atherosclerosis population. J Atheroscler Thromb.

[18] Wang MX, Jiao JH, Li ZY, Liu RR, Shi Q, Ma L (2013). Lutein supplementation reduces plasma lipid peroxidation and C-reactive protein in healthy nonsmokers. Atherosclerosis.

[19] McEneny J, Wade L, Young IS, Masson L, Duthie G, McGinty A (2013). Lycopene intervention reduces inflammation and improves HDL functionality in moderately overweight middle-aged individuals. J Nutr Biochem.

[20] Palozza P, Parrone N, Simone RE, Catalano A (2010). Lycopene in atherosclerosis prevention: an integrated scheme of the potential mechanisms of action from cell culture studies. Arch Biochem Biophys.

[21] Han H, Cui W, Wang L, Xiong Y, Liu L, Sun X (2015). Lutein prevents high fat diet-induced atherosclerosis in ApoE-deficient mice by inhibiting NADPH oxidase and increasing PPAR expression. Lipids.

[22] Ford ES, Gillespie C, Ballew C, Sowell A, Mannino DM (2002). Serum carotenoid concentrations in US children and adolescents. Am J Clin Nutr.

[23] Ford ES, Mokdad AH, Giles WH, Brown DW (2003). The metabolic syndrome and antioxidant concentrations: findings from the Third National Health and Nutrition Examination Survey. Diabetes.

[24] Sluijs I, Beulens JW, Grobbee DE, van der Schouw YT (2009). Dietary carotenoid intake is associated with lower prevalence of metabolic syndrome in middle-aged and elderly men. J Nutr.

[25] Gao Qin, Zhong Chunrong, Zhou Xuezhen, Chen Renjuan, Xiong Ting, Hong Miao, Li Qian, Kong Man, Han Weizhen, Sun Guoqiang, Yang Xuefeng, Yang Nianhong, Hao Liping (2019). The association between intake of dietary lycopene and other carotenoids and gestational diabetes mellitus risk during mid-trimester: a cross-sectional study. British Journal of Nutrition.

[26] Chan AW, Tetzlaff JM, Altman DG, Laupacis A, Gotzsche PC, Krleza-Jeric K (2013). SPIRIT 2013 statement: defining standard protocol items for clinical trials. Ann Intern Med.

[27] Xu X-R, Zou Z-Y, Xiao X, Huang Y-M, Wang X, Lin X-M (2013). Effects of lutein supplement on serum inflammatory cytokines, ApoE and lipid profiles in early atherosclerosis population. J Atheroscler Thromb.

[28] Yang YX, Wang GY, Pan XC (2009). China food composition table (2nd ed).

[29] United States Department of Agriculture and Agricultural Research Service. USDA Food Composition Database. http://adb.nal.usda.gov/ndb/nutrients/index. Accessed 11 June 2019.

[30] Shao A, Hathcock JN (2006). Risk assessment for the carotenoids lutein and lycopene. Regul Toxicol Pharmacol.

[31] Zou ZY, Xu XR, Lin XM, Zhang HB, Xiao X, Ouyang L (2014). Effects of lutein and lycopene on carotid intima-media thickness in Chinese subjects with subclinical atherosclerosis: a randomised, double-blind, placebo-controlled trial. Br J Nutr.

[32] Zou Z, Xu X, Huang Y, Xiao X, Ma L, Sun T (2011). High serum level of lutein may be protective against early atherosclerosis: the Beijing atherosclerosis study. Atherosclerosis.

[33] Kelli HM, Corrigan FE, Heinl RE, Dhindsa DS, Hammadah M, Samman-Tahhan A (2017). Relation of changes in body fat distribution to oxidative stress. Am J Cardiol.

[34] Andersen L, Jacobs DR, Gross MD, Schreiner PJ, Williams DO, Lee D-H (2006). Longitudinal associations between body mass index and serum carotenoids: the CARDIA study. Br J Nutr.

